# A New Instrument for Indicating the Movement of the Heart

**Published:** 1856-10

**Authors:** 


*A new Instrument for indicating the Movement of the Heart.*

Dr. Scott Alison has exhibited an instrument to the Royal Society, which he calls a sphygmoscope, and employs it to indicate the movements of the heart and blood vessels. The construction is simple: a small glass tube, about a foot in length, open at the upper end, and with a graduated ivory scale affixed, terminates below in a hemispherical or trumpet-mouth, bent to a right angle with a tube. This mouth is covered with a water proof membrane, and, being filled with colored water, is to be pressed against the ribs where the movement of the heart is most sensible. At once the water starts up the tube, in which it is seen to rise and fall in every beat; and thus all the movements of the vital organ, whether regular or irregular, may be distinctly viewed and measured by means of the scale. A smaller instrument of the same kind will show the beating of the pulse or of any other blood vessel, however small; and the beats may be compared with those of the heart. They are perceptible even at the end of an India rubber tube two feet in length. Already some new physiological conclusions have been arrived at with regard to the circulation of the blood, and a further insight into vital action is hoped for from the general use of the sphygmoscope among medical practitioners.—*Boston Med. and Surg. Journal.*

